# Intelligent Systems for Molecular Biology 2002 (ISMB02)

**DOI:** 10.1002/cfg.215

**Published:** 2002-12

**Authors:** K. Cara Woodwark

**Affiliations:** Biomolecular Sciences UMIST Manchester M60 1QD UK

## Abstract

This report profiles the keynote talks given at ISMB02 in Edmonton, Canada, by Michael Ashburner, Barry Honig, Isidore Rigoutsos, Ford Doolittle, Stephen Altschul, Terry Gaasterland, John Reinitz, and the Overton Prize winner, David Baker.


**Comparative and Functional Genomics**
*Comp Funct Genom* 2002; **3**: 484–486.
Published online in Wiley InterScience (www.interscience.wiley.com). **DOI:** 10.1002/cfg.215

**Feature**

### Meeting Review: Intelligent Systems for Molecular Biology 2002 (ISMB02)

3–7 August 2002, Shaw Conference Centre, Edmonton, Canada

K. Cara Woodwark*
*Biomolecular Sciences, UMIST, Manchester M60 1QD, UK*

**Correspondence to:*
*K. Cara Woodwark, Biomolecular Sciences, UMIST, Manchester M60 1QD, UK.*
*E-mail:*
*Cara.Woodwark@astrazeneca.com*

Received: 8 October 2002
Accepted: 8 October 2002

### Abstract

**This report profiles the keynote talks given at ISMB02 in Edmonton, Canada, by Michael Ashburner, Barry Honig, Isidore Rigoutsos, Ford Doolittle, Stephen Altschul, Terry Gaasterland, John Reinitz, and the Overton Prize winner, David Baker. Copyright © 2002 John Wiley & Sons, Ltd.**

**Keywords:** gene ontologies; electrostatics; pattern discovery; phylogenetic trees; sequence search algorithms; mouse gene analysis; spatial expression patterns; protein structure prediction

### Introduction

As ISMB01 in Copenhagen had been voted an overwhelming success, Edmonton had much to live up to. The cold and damp weather, combined with jetlag for the European contingent, tried to dampen spirits but to no avail, and a very full and interesting conference was enjoyed by all.

Only the keynote talks are reported here, as the abstracts of all the posters are freely available online at: **http://www.ismb02.org/poster.html** and those of the papers are available at: **http://bioinformatics.oupjournals.org/content/vol18/suppl_1/index.shtml**

### Keynote talks

The opening speaker was the indomitable **Michael Ashburner** (European Bioinformatics Institute and Cambridge University, UK), who introduced **gene ontologies** as an important backbone upon which to build integrated systems — we need to be able to communicate between disciplines and organisms in ways that scientists and computers can understand, and having common terms of reference will greatly facilitate this. Indeed, the use of gene ontologies to simplify communication formed a common undercurrent in many subsequent talks.

**Barry Honig** (Columbia University) gave a fascinating talk on how we can greatly increase our understanding of protein function by explaining protein–protein, protein–DNA and even protein–membrane **interactions in terms of electrostatics**. We may know the structure of a protein but this will not necessarily show the position of the functional site; however, once the electrostatics of the molecule are modelled, the functional site often becomes obvious. He also introduced one of the other underlying themes of the conference; the use of phylogenetics to increase our understanding of biological systems. For example, he recommended that the phylogenetic tree of a protein family of interest should be drawn so that it is possible to decide whether a structure/sequence is conserved because it has function or whether the sequence is apparently conserved because not enough time has elapsed for the sequences to have diverged.

**Isidore Rigoutsos** (IBM) gave an interesting talk on **pattern discovery in data mining**. He described two viable approaches: bottom-up, i.e. start with a pattern and see where it occurs; or top-down, i.e. start with data and look for patterns. He described a hybrid method, **Teiresias**. This intriguing-sounding method may be used for

Copyright © 2002 John Wiley & Sons, Ltd.

anything that can be construed as an alpha-numeric stream, such as sequence (DNA or protein), expression patterns, or secondary structure motifs ($\alpha$-helix, $\beta$-sheets, etc.) This method has even been used to detect ftp intruders with a 100% success rate. The methodology is apparently very similar to that of Michael Ventris, the man who in 1952 deciphered the linear B texts of the Mycenaeans. It involves looking for patterns of length $l$, found $k$ times with an interval of $w$. Overlaps between patterns are then looked for, to elucidate the maximum size of each pattern. Isidore has a website where alpha-numeric data may be inputted to use Teiresias to look for any patterns stored in the data stream: **http://cbcsrv.watson.ibm.com/Tspd.html**

**Ford Doolittle** (Dalhousie University) gave a fascinating talk on the importance of **drawing phylogenetic trees with both genes and genomes**. He highlighted the need to draw phylogenetic trees from protein-coding genes, rather than relying completely on the rRNA tree for the species involved, as the protein trees would give a more complete insight into the true evolutionary relationships between organisms. This is especially true as horizontal transfer has been found in all organisms except higher eukaryotes (although it can not be completely ruled out in these) and can cause some proteins in an organism to have a different evolutionary history than some of the other proteins. Even some ribosomal genes have been found to be horizontally transferred.

He also spoke of the efforts to elucidate the core set of genes that would have been found in the common ancestor of all living things. Unfortunately, this seems to be an impossible task as, strangely, there does not appear to be a core set of genes, although the situation is confused by the immense amount of gene sharing — horizontal transfer — of genes that has occurred in lower organisms.

**Stephen Altschul**, of NCBI BLAST fame, gave a talk on **the use of ROCs (Receiver Operator Character curves)**, based on the area under the graph of false positives vs. true positives, to compare the sensitivity and selectivity of different sequence search algorithms or the same algorithm with different parameters. Significance could be assigned by bootstrapping. He also noted that ROCs demonstrated the improvement to PSI-BLAST. This new version of PSI-BLAST makes use of compositional-based statistics, in that the proportions of amino acids may vary between sequences from different organisms due to GC content, etc.

Topical as ever, **Terry Gaasterland** (Rockefeller University) spoke about **an analysis of mouse genome data** that had been released publicly just 2 days previously. She described the increased knowledge that is being gained of the complex nature of genes — with alternative splice sites, different stops and starts, and how the inclusion (or exclusion) of a particular exon can have a great affect on the translation start site, even though it is downstream of that exon. Many of the genes that may skip exons also change the frame of the translation if the exon is missed, thus creating a completely different protein product! Which begs the question — can we really call this the same gene?

Terry also described the comparison of mouse genes upregulated under a particular condition with the same genes in humans. The methodology involved BLASTing the upstream 100 kb from the mouse and human genes against each other and looking for known motifs. If enough motifs were found above a certain threshold (as motifs can occur in common by chance), then the genes would be tested in the lab to see if they were indeed under similar regulatory mechanisms, i.e. co-regulated.

Her take-home message was that, in bioinformatics, we should start with biology, add in the computational predictions and then go back and test them in the lab. This re-coupling of the wet/dry cycle is the best hope we have of deciphering the mountain of data we now have at our disposal.

**John Reinitz** (SUNY), in his keynote address on **elucidating spatial expression patterns in *Drosophila***, also stressed the need for wet lab and *in silico* biology to be combined in order to understand the full range of biological functions. He explained an interesting system, called the 'gene circuit method' that allows the expression of genes in the *Drosophila* blastoderm to be modelled. This involves the formation of a theoretical model, followed by the repeated visualization of three different genes of the 14 genes that are expressed at this stage, using fluorescently-tagged antibodies, the results of which are compared to elucidate the regulatory pathways involved.

**David Baker** (University of Washington, Seattle) presented the Overton prize lecture on **the**

Copyright © 2002 John Wiley & Sons, Ltd.

**prediction and design of protein structure**. He is also the winner of CASP4, where teams from around the world compete to predict as accurately as possible the structure of a protein for which the known structure is held as a closely guarded secret. He gave a fascinating talk that showed that protein structure prediction is finally coming of age, although he would be the first to admit that they are still not close enough for drug discovery or design. However, they have been able to create proteins with lower folding energies than the wild-type protein, or to change the way a fold works to create dimers from monomeric proteins. This came about in an effort to be able to create proteins that will specifically interact with others. When they design these proteins from scratch they have discovered that they end up with a sequence that is very similar to the original wild-type protein. Baker also introduced us to the CAPRI competition, where they will be given the structure of a target and corresponding enzyme and have to work out how they interact. Perhaps Barry Honig could give him some hints and tips there.

### Conclusions

ISMB02 proved that bioinformatics is still going from strength to strength and that one of the key developmental areas is the integration of data and techniques. This conference is one of the best places to learn of new data, new techniques and, perhaps more importantly, the novel use of old tried and tested techniques.

The next ISMB is to be in Brisbane, Australia, but there was a lot of debate in Edmonton as to whether the full range of satellite meetings (Bioinformatics Open Source Conference, Bio-ontologies, Biopathways, and the Workshop on Education in Bioinformatics) would be held either side of the meeting, as has been the case over the past few years, or whether some of these might not become conferences in their own right. Therefore, it may be a different-looking ISMB, as some of the fledglings fly the nest in the next couple of years. Still, surf's up! Now where did I put the insect repellent and the sun cream?

Copyright © 2002 John Wiley & Sons, Ltd.

